# Reflections on DeepSeek's breakthrough

**DOI:** 10.1093/nsr/nwaf044

**Published:** 2025-02-12

**Affiliations:** Scientific Director, Institute of Neuroscience, Center for Excellence in Brain Science and Intelligence Technology, Chinese Academy of Sciences, China

The year 2025 will be one of the most memorable years for Chinese science in recent decades. On 20 January, a small AI company, DeepSeek, in Hangzhou announced an incredibly efficient ‘reasoning’ AI model named DeepSeek R1 that sent shockwaves around the globe, prompting some enthusiasts to call it an ‘AI revolution’. The shock comes from the fact that DeepSeek R1 rivals the top large language models (LLMs) such as ChatGPT-o1 produced by giant AI companies, but R1’s base LLM V3 (announced a few weeks earlier) was built with a small fraction of the cost and used a much smaller number of low-grade computer chips (GPUs) than the current top LLMs. Most remarkably, DeepSeek's models are open source, with full disclosure of technical details, and AI developers are charged a much lower fee than for other closed-source LLMs on the market. As the shock now subsides, we may reflect upon the real significance of this DeepSeek breakthrough.

DeepSeek R1 made very clever use of existing network tools, such as a mixture of experts, reinforcement learning and data distillation, to achieve a totally unexpected reasoning efficiency. For the first time, it reveals the ‘chain-of-thought’ of its reasoning process to the user. However, R1 does not involve any groundbreaking invention of new AI technology in the same sense as the back-propagation algorithm and transformer network that respectively brought the two recent AI revolutions—deep learning and LLMs. In popular jargon, it is not a 0-to-1 invention, but a 1-to-100 development with unprecedented speed. This speed announces the imminent arrival of artificial general intelligence (AGI)—the self-learning and generalization capability of machines to perform a wide variety of tasks in complex environments, an intelligence that matches or even surpasses human intelligence. Importantly, DeepSeek showed clearly that 1-to-100 development could have a more significant impact than a 0-to-1 invention.

DeepSeek's LLMs appeared at a time when the AI community was largely persuaded by the scaling law, i.e., the performance of AI models improves with increasing model size, dataset and computing power. Despite some evidence indicating that this law is reaching its limit, many tech giants in the USA and China are still demanding that investors and governments support larger and larger data centers with energy consumption that is non-sustainable for societies and the environment. Construction of many big data centers is ongoing and the US president just announced US$500 billion in support for such an endeavor. DeepSeek R1 makes a strong pitch that improving the efficiency of computing algorithms could be a much more attractive approach than adhering to the scaling law.

DeepSeek's ‘wake-up call’, as President Trump put it, is not just that Chinese AI technology is rapidly catching up in the USA–China AI race. It is also a call to the global AI community that the future of AI is not just in the hands of a few tech giants. Small groups of researchers could play an important role in pushing the AI frontier. The real AI race is now between the open-source culture of the global AI community and the profit-driven closed-source culture of AI tech companies. The ‘democratization’ of AI technology is perhaps the most significant aspect of the DeepSeek breakthrough.

DeepSeek also made a wake-up call to Chinese research institutions, which have acquired large amounts of government funding for AI and employed the great majority of Chinese AI research talent. Despite the amazing increase in the number of research papers published by Chinese AI researchers in recent years, truly impactful discovery or invention in the AI field continues to be scarce, and the culture of quick publication of papers with incremental value prevails. The leader of DeepSeek, Liang Wenfeng, has unremarkable academic credentials and was rarely invited to speak in academic AI meetings, up to the announcement but he has the confidence and audacity to tackle the most important problem of developing AGI. The wake-up call is both alarming and inspiring for all young Chinese scientists and engineers striving to make a difference in the world.

The key to the innovative culture of DeepSeek, as Liang recently explained, is to instill confidence and bottom-up initiatives in his research team. Small teams like that of DeepSeek are more efficient for rapid closed-loop interaction, for cohesive efforts towards defined goals, and for the spiritual bonding of team members. Most Chinese institutions leave little room for young people at the bottom of the hierarchy to pursue their own ideas. Avenues for unleashing the innovative talents of young researchers, such as supporting extraordinarily talented fresh PhDs to set up independent research labs without doing postdoctoral training, have been demonstrated by the successful examples of MIT's Whitehead Fellows Program and Young Investigators Program at the Institute of Neuroscience of the Chinese Academy of Sciences.

The DeepSeek breakthrough, despite its innovativeness, is still within the framework of LLMs that currently dominates the AI field. As the demand for computing power reaches its limit, further development of LLMs will depend on more efficient computation algorithms and network structures. Most AI researchers now pay little attention to how the brain works, since LLMs seem so much more powerful than the human brain in many aspects, and opportunities for LLM applications abound. Nevertheless, as AGI is becoming a closer target, we may have to look more seriously at how the brain's highly efficient computation is achieved, and how human intelligence is embodied in a system that can effectively interact with the outside world [1].

Development of embodied AGI in physical systems such as human-like robots requires close interdisciplinary interaction among software engineers, bioengineers and neuroscientists. The task seems quite clear: a language-based reasoning and decision-making LLM needs to be interfaced on the input end with integrated perception of multiple sensory signals, including language-based instructions, and on the output end with sophisticated motor systems for executing behaviors. The five-year plan for the second phase of the China Brain Project, known as the 2030 big S&T project ‘Brain Science and Brain-Inspired Technology’, is now on the drawing board. Within the next five years, we look forward to seeing close interactions between neuroscientists and AI researchers for the realization of embodied AGI that not only achieves human-level intelligence but also aligns with human needs.

***Conflict of interest statement*.** None declared.

## Notes

Executive Editor-in-Chief NSR
